# Physical and Chemical Barriers in Root Tissues Contribute to Quantitative Resistance to *Fusarium oxysporum* f. sp. *pisi* in Pea

**DOI:** 10.3389/fpls.2018.00199

**Published:** 2018-02-19

**Authors:** Moustafa Bani, Alejandro Pérez-De-Luque, Diego Rubiales, Nicolas Rispail

**Affiliations:** ^1^Institute for Sustainable Agriculture, Consejo Superior de Investigaciones Científicas, Córdoba, Spain; ^2^Ecole Nationale Supérieure de Biotechnologie, Constantine, Algeria; ^3^Área de Mejora y Biotecnología, IFAPA, Centro Alameda del Obispo, Córdoba, Spain

**Keywords:** cell wall strengthening, disease resistance, *Fusarium oxysporum*, papilla-like structure, phenolic compounds, *Pisum sativum*, quantitative resistance, resistance mechanism

## Abstract

Fusarium wilt caused by *Fusarium oxysporum* f. sp. *pisi* (*Fop*) is one of the most destructive diseases of pea worldwide. Control of this disease is difficult and it is mainly based on the use of resistant cultivars. While monogenic resistance has been successfully used in the field, it is at risk of breakdown by the constant evolution of the pathogen. New sources of quantitative resistance have been recently identified from a wild relative *Pisum* spp. collection. Here, we characterize histologically the resistance mechanisms occurring in these sources of quantitative resistance. Detailed comparison, of the reaction at cellular level, of eight pea accessions with differential responses to *Fop* race 2, showed that resistant accessions established several barriers at the epidermis, exodermis, cortex, endodermis and vascular stele efficiently impeding fungal progression. The main components of these different barriers were carbohydrates and phenolic compounds including lignin. We found that these barriers were mainly based on three defense mechanisms including cell wall strengthening, formation of papilla-like structures at penetration sites and accumulation of different substances within and between cells. These defense reactions varied in intensity and localization between resistant accessions. Our results also clarify some steps of the infection process of *F. oxysporum* in plant and support the important role of cell wall-degrading enzymes in *F. oxysporum* pathogenicity.

## Introduction

*Fusarium oxysporum* Schl. f. sp. *pisi* Snyd. and Hans. (*Fop*) is an important pathogen causing vascular wilt of field pea (*Pisum sativum* L.) worldwide (Rubiales et al., 2015). Four different races of *Fop*, named 1, 2, 5, and 6, have been described so far (Infantino et al., 2006). Races 1 and 2 are widely distributed, while races 5 and 6 are, to date, only important in western Washington State (Infantino et al., 2006). Control of soil-borne fungal diseases is mainly achieved by the integration of different disease management procedures. Among these methods, the use of resistant cultivars is widely recognized as the safest, most economical and most effective crop protection method (Ciancio and Mukerji, 2008; Rubiales et al., 2015). Resistance to *Fop* races 1, 5, and 6 is conferred by single dominant genes whereas resistance to race 2 is quantitative (Bani et al., 2012; McPhee et al., 2012).

Similarly to other *formae speciales* of *F. oxysporum*, *Fop* behaves as a classical soil-borne pathogen with propagules that can survive in soil during extended periods of time in the absence of suitable host (Roncero et al., 2003). The infection cycle of *F. oxysporum* is initiated by spore germination and its directed elongation toward host plant root in response to specific plant signals (Nelson, 1991; Turrà et al., 2015). Then the growing, infective hyphae adhere to host roots and penetrate it through wounds or by piercing through the epidermis (Nelson, 1981; Bishop and Cooper, 1983a; Benhamou and Garand, 2001; Zvirin et al., 2010). The mycelium then advances intercellularly through the root cortex, until it reaches the xylem vessels and colonize them through the pits (Bishop and Cooper, 1983b; Beckman, 1987). Then, the fungus progresses vertically, through xylem vessels, to invade the shoot (Bishop and Cooper, 1983b; Beckman, 1987). After wilting and plant death, the fungus reaches the plant surface where it produces chlamydospores that are dispersed onto the soil for a second cycle of infection (Kraft et al., 1994). During root invasion and colonization, *Fop* is exposed to various plant defense mechanisms (Beckman, 1987; Michielse and Rep, 2009).

Several histological studies have characterized the infection processes and resistance mechanisms in different plant species (Bishop and Cooper, 1983a,b; Beckman, 1987; Baayen et al., 1989; Tessier et al., 1990; Pereira et al., 2013). Successful host invasion by *F. oxysporum* depends on a multitude of factors, which vary according to the pathosystem. Similarly, hosts develop different physical and chemical barriers to block the pathogen progressions at different levels depending on host species or cultivar. The most efficient mechanisms are production of antifungal compounds, formation of papillae at penetration sites, suberization and lignification of cell walls, accumulation of gums, gels or tyloses within xylem cells and vessel crushing by proliferation of adjacent parenchyma cells (Bishop and Cooper, 1983a; Baayen and Elgersma, 1985; Beckman, 1987; Charchar and Kraft, 1989; Ouellette et al., 1999; Grayer and Kokubun, 2001; Yadeta and Thomma, 2013; Pouralibaba et al., 2017). These mechanisms contributes to block pathogen progression and to kill invading hypha. Although several of these mechanisms are often described simultaneously within host root, their relative contribution to resistance varies depending on the host and cultivar. In pea, previous reports demonstrated that the main determinant of resistance to *Fop* was the occlusion of xylem cells and to a lesser extent to xylem cell wall strengthening (Bishop and Cooper, 1983a, 1984; Tessier et al., 1990; Benhamou and Garand, 2001). Interestingly, occluding pea cells accumulated gels composed of a mixture of carbohydrates, proteins and pectins instead of tylose commonly detected in other *F. oxysporum-*host interaction (Bishop and Cooper, 1984; Tessier et al., 1990). Papilla formation at epidermis and cortex, and endodermis suberization was also reported in pea. However, they were shown to play only little role in resistance against *Fop*, if any, since they were also detected in susceptible accessions (Bishop and Cooper, 1983a).

Recently, new sources of quantitative resistance have been identified in pea and *Medicago truncatula* (Bani et al., 2012; Rispail and Rubiales, 2014). Although some reports have described the histopathology of resistant and susceptible pea cultivars (Bishop and Cooper, 1983a,b; Tessier et al., 1990), detailed studies of the infection process and of the resistance mechanisms of genotypes with different levels of resistance are scarce. In this work, we aimed to determine in detail the infection process of *Fop* race 2 in eight pea accessions showing different levels of quantitative resistance and to characterize the resistance mechanisms established in these accessions.

## Materials and Methods

### Fungal Isolate and Cultural Conditions

*F. oxysporum* f. sp. *pisi* (*Fop*) race 2 strain R2F42, kindly provided by Dr. W. Chen (USDA-ARS, Pullman, WA, United States), was used in all experiments. The fungal strain was stored as microconidial suspensions at -80°C in 30% glycerol. For microconidia production, cultures were grown in potato dextrose broth (Difco, Detroit, MI, United States) at 28°C under constant shaking at 170 rpm. *Fop* inoculum was prepared by filtering and centrifuging 4-day-old PDB cultures and adjusting the conidia to 5 × 10^6^ *Fop* conidia ml^-1^ in sterile distilled water with a haemocytometer.

### Plant Materials, Growth Conditions and Inoculation

Eight *Pisum* spp. accessions with a wide range of responses to *Fop* race 2 were used, including the susceptible accessions P21 (PI505059) and P629 (IFPI2353), the partially resistant accessions JI2480 and Messire and the resistant accessions P42 (PI268480), P633 (IFPI2357), JI1412 and JI1760 (**Table 1**) (Bani et al., 2012).

**Table 1:** List of *Pisum* ssp. accessions used in this study.

Accession	Synonym Code	Plant Species	Reaction to *Fop R2*
JI1412		*P. sativum* ssp. *sativum*	R
JI1760		*P. sativum* ssp. *sativum*	R
P633	IFPI2357	*P. sativum* ssp. *arvense*	R
P42	PI268480	*P. sativum* ssp. *elatius*	R
JI2480		*P. sativum* ssp. *sativum*	PR
Messire		*P. sativum* ssp. *sativum*	PR
P629	IFPI2353	*P. sativum* ssp. *arvense*	S
P21	IFPI2495	*P. sativum* ssp. *elatius*	S


*Disease reaction to *Fop* race 2 has been defined according to Bani et al. (2012).*

Pea seeds were surface-sterilized in 20% bleach solution and germinated, in the dark, wrapped in wet filter paper in petri dishes as described previously (Bani et al., 2012). Germinated seeds were, then, sown in vermiculite and grown in controlled environment under a 16/8 h light-dark photoperiod at 26 ± 2°C with 200 μmol m^-2^ s^-1^ irradiance. Plants were watered every 3 days with tap water. Seven-day-old pea seedlings were inoculated with *Fop* inoculum (5 × 10^6^ *Fop* conidia ml^-1^) using the root dipping technique with root trimming as described previously (Bani et al., 2012). For this, plant roots were submerged in the *Fop* inoculum for 5 min. Control plants were similarly trimmed but dipped in sterile distilled water. To study the influence of root trimming on the infection process, additional pea seedlings were also inoculated without root trimming (Rispail et al., 2015). In this case, the whole root system of each plant was immersed in the inoculum for 15 min. Inoculated and control plants were maintained in the same growth conditions as above. A total of 10 plants were used per accession and experiment. The experiments were repeated thrice independently. In each independent experiment, six plants were collected to perform the histological study, one plant was collected to isolate *Fop* colonies and confirm plant inoculation and three plants were maintained in the growth chamber to follow the progression of disease symptoms macroscopically. Disease severity was estimated at 30 dpi by calculating the percentage of leaves showing symptoms per plants as described previously (Bani et al., 2012).

### Sampling and Fixation of Plant Tissues

Root and stem tissues from three plants per accession were sampled at 4 or 7 days post-inoculation (dpi). For each plant, 5–6 mm long segments of roots and stems were fixed. Two procedures were used to fix and section plant samples: (i) the sampled material was fixed in FAA solution (50% ethanol, 5% formaldehyde, 10% glacial acetic acid, in water) for 48 h. Fixed samples were then dehydrated in ethanol series (50, 80, 95, 100, 100% for 12 h each) and transferred to xylene (Merck KGaA, Darmstadt, Germany) through xylene-ethanol series (30, 50, 80, 100, 100% xylene for 12 h each) and finally infiltrated and saturated with paraffin (Paraplast plus^®^; Sigma, Switzerland) prior to embedding and preparing the paraffin blocks. Seven μm-thick sections were then cut with a rotary microtome (Nahita 534; Auxilab SA, Beriain, Spain) and attached to adhesive-treated microscope slides (polysine slides; Menzel GmbH and Co KG, Braunschweig, Germany). (ii) The sampled material was fixed in Karnovsky solution (5% glutaraldehyde and 4% formaldehyde in 0.025 M cacodylate buffer complemented with 0.5 mg ml^-1^ of CaCl_2_) (Karnovsky, 1965) for 4 h at room temperature. Fixed samples were washed three times with 0.025 M cacodylate buffer for 20 min each, and dehydrated in ethanol series (50, 80, 95% for 12 h each). Samples were then embedded in Leica Historesin (Leica Microsystems Nuβloch GmbH, Heidelberg, Germany). Semi-thin sections (2 μm) were cut with a semi-automated rotary microtome (Leica RM2245, Leica microsystems, Germany) and attached to microscope slides.

### Cytochemical Methods for Light and Epifluorescence Microscopy

After paraffin removal, FAA-fixed sections were stained with a mixture of alcian green:safranin (AGS) (Joel, 1983). The slides were dried and mounted with DePeX (BDH). With this staining method, carbohydrates (including cell walls and mucilages) appeared green, yellow or blue, while lignified, cutinized and suberized walls, as well as tannin and lipid material inside cells appeared red (Joel, 1983). Semi-thin (2 μm) histo-resin sections of Karnovsky-fixed samples were stained with 0.1% toluidine blue O (TBO) in citrate buffer (pH 5) for 5 min. The TBO staining method allows detection of phenolics including tannin, lignin and suberin (Baayen et al., 1996; Crews et al., 2003). The sections were observed using a light microscope (Leica DMLB, Leica Microsystems Wetzlar GmbH, Wetzlar, Germany) and photographed using a digital camera (Nikon DXM1200F, Japan). The samples were also observed by epi-fluorescence under excitation at 450–490 nm (blue-violet) with the same microscope.

### Data Analysis

At least three paraffin- and resin-embedded blocks from independent biological replications were stained, sectioned and visualized for each genotype, tissue and treatment per independent experiment. For each block, 10 representative sections were selected randomly and visualized. The relative frequency of each defense reaction was thus estimated on a total of 90 root sections from 9 independent blocks and expressed as percentage per root section. A reaction detected in less than 10% of the thin-sections was considered rare. A reaction detected in 10–30% of the thin-sections was considered low while it was considered frequent when observed in 30–60% of the sections. A reaction detected in more than 60% of the thin-sections was considered highly frequent.

All experiments followed a complete randomized design. For ease of understanding, means of raw percentage data are presented in Tables and Figures. However, for statistical analysis, data recorded as percentages were subjected to angular transformation to normalize data and stabilize variances (Baird et al., 2002) before being subjected to analysis of variance using SPSS software. Afterward, residual plots were inspected to confirm data conformed to normality. Shapiro–Wilk test and Bartlett’s test was performed to test normality and homogeneity of variances respectively. Significance of differences between means was determined through multiple comparisons according to Tukey’s range test. For multivariate analyses, data were analyzed using canonical variate analysis. Pearson correlations were calculated to detect statistical correlations between resistance responses and the outcome of fungal colonization.

## Results

### Macroscopic Events

Confirming previous results, accessions P21 and P629 were highly susceptible (Supplementary Figure S1). In these accessions, the initial symptoms were detected at 10 dpi with the yellowing of the primary leaf margins. This yellowing progressively reached the interveinal areas often associated with leaf necrosis until the whole leaf wilted and die. Simultaneously, these symptoms sequentially reached the later formed leaves until the whole plant withered and died within 21 days. The partially resistant accessions Messire and JI2480 remained alive throughout the experiment but showed high disease severity since 76.5 and 62.97% of their leaves showed typical wilt symptoms at 30 dpi respectively. In turn, the phenotype of the inoculated resistant accessions (P42, JI1412, JI1760 and P633) remained similar to non-inoculated control plants throughout the experiment. No significant differences in disease severity were detected between inoculation methods (*p* = 0.844).

To confirm that all plants had been in contact with *Fop* inoculum, *Fop* colonies were re-isolated from inoculated plants at 7 dpi. As expected from previous studies (Bani et al., 2012), *Fop* colonies were detected in root fragments of all accessions. However, fungal colonies were only recovered from stem fragments of susceptible accessions indicating that as early as 7 dpi, *Fop* had already colonized the whole plant in these accessions (Supplementary Figure S2).

### Histopathology of Fop in Pea Plants

**FIGURE 1:**
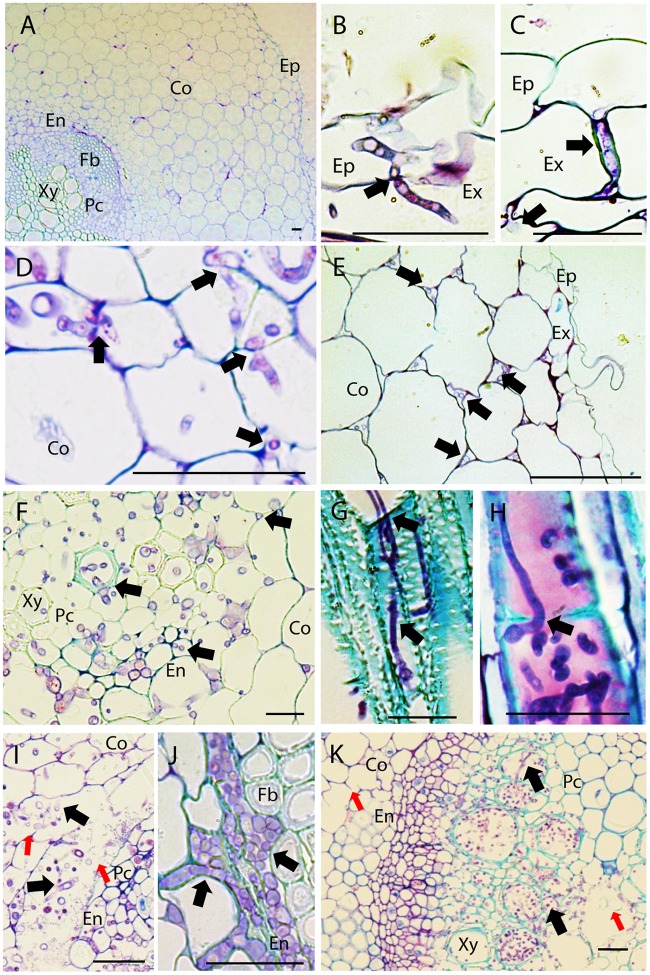
Histopathology of *Fusarium oxysporum* f. sp. *pisi* (*Fop*) in pea accessions. The figure represents longitudinal and cross-sections of pea roots inoculated or not with *Fop* race 2 stained with TBO. **(A)** Cross-section of the susceptible P21 root maintained non-inoculated, showing a general view of pea root histology: epidermis, (Ep) cortex (Co), endodermis (En), parenchyma cells (Pc), Fiber cells (Fb), and xylem vessels (Xy). **(B)** Longitudinal section of *Fop-*inoculated root of the resistant accession P633 at 4 dpi, showing the penetration of an exodermal cell (Ex) by an infective hypha. Note the constriction of the hypha at site of cell wall penetration (black arrow). **(C)** Cross-section of the partially resistant accession Messire root at 7 dpi, showing the root penetration by *Fop* hypha growing between two exodermal cells (black arrow). **(D)** Cross-section of susceptible P629 roots at 7 dpi, showing intra- and intercellular progression of infective hyphae (Black arrows) through cortex and endodermis. Note the constriction of hypha at site of cortical cell penetration (Black arrows). **(E)** Cross section of Messire root at 7 dpi showing intercellular progression of infective hyphae through root cortex (Black arrows). **(F)** Cross-section of P21 root at 7 dpi after inoculation without root trimming showing abundant colonization of vascular tissue. **(G)** Longitudinal section of susceptible P21 root at 7 dpi showing inter- and intra-tracheary colonization of xylem vessels by elongation of infective hyphae (Black arrow). **(H)** Longitudinal section of resistant accession P633 at 7 dpi showing intra-tracheary colonization of xylem vessel by *Fop* conidia germinating through xylem perforation plate (Black arrow). **(I)** Cross section of the root of the susceptible accession P629 at 7 dpi showing intense degradation of endodermal cell layer (red arrows) and the abundant *Fop* development in the created intercellular space (Black arrows). **(J)** Cross-section of the partially resistant Messire root at 7 dpi showing abundant colonization of the intracellular space of endodermal cells by *Fop* (Black arrows). **(K)** Cross-section of the susceptible P21 hypocotyl showing abundant colonization of xylem vessels by *Fop* (Black arrows) and cell degradation in presence or absence of fungal structure (Red arrows). Bar = 25 μm.

Sections of non-inoculated control pea roots presented the characteristic structure of dicotyledonous roots (**Figure 1A**). Four dpi, inoculated pea roots were already extensively colonized by *Fop*. At this stage, infective hyphae had already penetrated the epidermal cells, colonized the cortex, and reached the vascular system. In all accessions, infective hyphae entered host root by penetrating into epidermal and exodermal cells (**Figure 1B**). *Fop* also penetrated pea roots by growing between epidermal or exodermal cells (**Figure 1C**). Specialized appresoria-like structures were never observed. Instead, the penetrating hypha appears to constrict itself to breach through the epidermal cell wall, restoring its normal size thereafter (**Figure 1B**). Once inside susceptible host root, fungal hyphae progressed through cortex and endodermis both inter- and intracellularly. At this stage, cell-to-cell infection by constricted hypha is often observed (**Figure 1D**). By contrast, fungal progression through root cortex of resistant accessions was exclusively intercellular (**Figure 1E**).

At 7 dpi, an abundant inter- and intracellular growth of fungi was observed in the endodermis, pericycle, fiber cells, parenchyma cells and xylem vessels of susceptible and partially resistant accessions even when plants were inoculated without root trimming (**Figure 1F**). By contrast, xylem vessels of resistant accessions were only colonized following root trimming inoculation (Supplementary Figures S3A,B). Within xylem vessel, the fungus developed intensively. Intra-tracheary colonization was mainly mediated by elongation of infecting hyphae although oriented germination of conidia through perforation plates was occasionally observed (**Figures 1G,H**). Simultaneously, colonization of vascular vessels was observed by infection of adjacent xylem vessels and parenchyma cells through cell-to-cell penetration by elongating hypha (**Figure 1G**). In susceptible accessions, the mycelial growth was associated with the extensive degradation of vascular parenchyma, fiber and cortical cells surrounding endodermis (**Figure 1I**). This created large intercellular spaces in which the pathogen developed intensively (**Figure 1I**). Cell degradation was not detected in resistant or partially resistant accessions despite the abundant accumulation of *Fop* cells in the endodermal intercellular space of partially resistant accessions (**Figure 1J**).

At 7 dpi, the fungus was also detected in the hypocotyl of susceptible accessions. Hypocotyl colonization was restricted to vascular tissue and associated with degradation of xylem parenchyma cells (**Figure 1K**). The pathogen was never detected in stems of resistant and partially resistant accessions (Supplementary Figure S3C).

### Histology of Resistance to Fop

**FIGURE 2:**
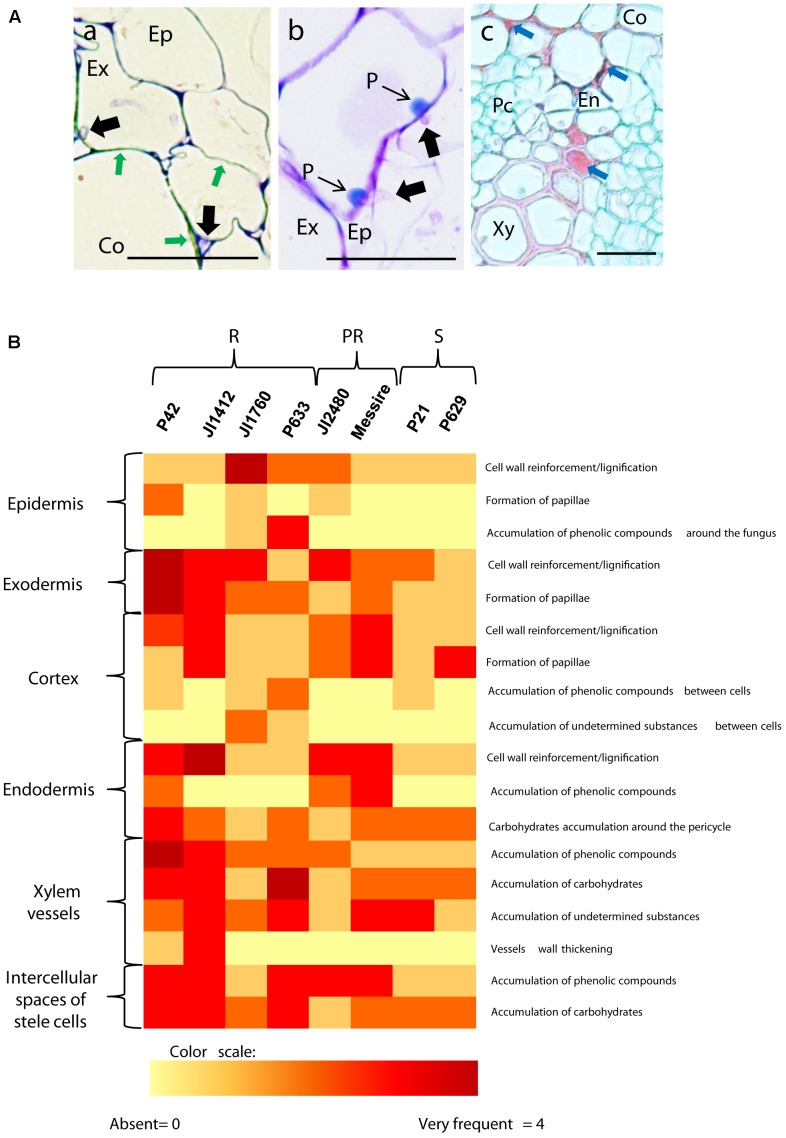
Description and frequency of resistance mechanisms detected in pea cells. **(A)** Cross sections of inoculated pea roots of resistant accessions illustrating the main mechanisms of resistance detected in pea. (a) TBO stained cross-section of inoculated root of the resistant P42 at 7 dpi showing the strengthening of cortical cell wall through lignification (Green arrows). (b) TBO stained cross-section of inoculated root of the resistant P42 at 7 dpi showing the apposition of defensive papilla preventing cell penetrations. (c) AGS stained cross-section of inoculated root of the resistant accession P42 at 7 dpi showing accumulation of undetermined substance in the intercellular space and within cells (Blue arrows). Black arrows indicate fungal cell presence. Bars = 25 μm. Ep, epidermis; Ex, exodermis; En, endodermis; Co, cortex; Xy, xylem cells; Pc, parenchyma cells. **(B)** Heatmap summarizing the frequency of host cell reactions observed 7 days after *Fop* inoculation in the different accessions at each root tissue. Intensity range from yellow (0 = absent reaction) to dark red (4 = very frequent) according to a visual scale ranging from 0 to 4 with 0 = absence of reaction, 1 = rare: reaction observed in less than 10% of the analyzed sections, 2 = low frequency: <30%, 3 = frequent: <60% and 4 = high frequency: >60%. R, resistant accessions; PR, partially resistant accessions; S, susceptible accessions.

Observation of the histological samples inoculated for 4 or 7 dpi indicated that pathogen progression was efficiently blocked in resistant and partially resistant accessions by the establishment of three mechanisms: cell wall strengthening through lignification, papilla formation and accumulation of (poly)phenolic and carbohydrates compounds (**Figure 2A**). Susceptible plants also developed some of these reactions as an attempt to counteract the massive pathogen infection, such as the formation of papilla at some sites of penetration or cell wall lignification. However, the pathogen overcame these defensive reactions and colonized the stele of susceptible accessions. Differences were detected between accessions regarding the frequency and localization in which these mechanisms were displayed. **Figure 2B** summarizes these differences according to accession and tissue type, using a visual/color scale established to estimate the frequency of each reaction ranging from 0 (yellow) to 4 (intense red). Resistant accessions such as P42 or JI1412 showed a high frequency (red color) in most of the observed defensive responses, whereas susceptible ones including P629 mostly showed a low frequency or absence (yellow). Partially resistant accessions presented intermediate values (**Figure 2B**). Given the diversity of responses detected between resistant accessions, we describe below the most relevant results for each root zone.

#### Outer Root Tissues (Epidermis and Cortex)

**FIGURE 3:**
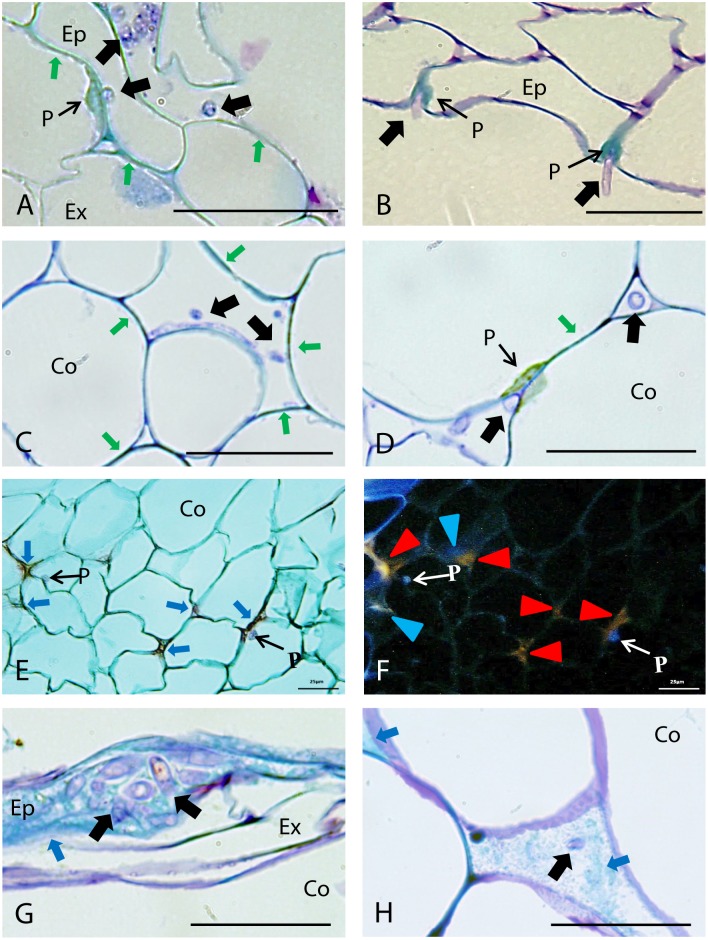
Defense responses and pathogen infection observed in root epidermis and cortical tissues of resistant pea accessions after inoculation with *Fop* race 2. Arrows indicate fungus. **(A)** Longitudinal section of the partially resistant Messire stained with TBO at 4 days post-inoculation (dpi) showing cell wall lignification (Green arrows) at epidermis (Ep) and the formation of a papilla at the exodermis (Ex) to prevent *Fop* hypha penetration. **(B)** TBO-stained cross-section of the resistant accession P42 at 4 dpi showing formation of papillae (P) in the epidermis at site of attempted fungal penetration. **(C)** Cross-section of the partially resistant accession Messire at 4 dpi, stained with TBO, showing the lignification of cortical cell walls (Green arrows) to prevent hypha penetration into cortical cells (Co). **(D)** TBO-stained cross-section of resistant accession JI1412 roots at 4 dpi showing the lignification of cortical cell wall (Green arrows) and formation of papillae (P) impeding fungal entry into cortical cells, although the fungi can progress through the cortex intercellularly (Black arrows). **(E)** Cross-section of the resistant accession JI1760 roots at 7 dpi, stained with alcian green:safranin mixture (AGS), showing papillae and the accumulation of undetermined substances (red staining indicated by Blue arrows) in the intercellular space of cortical cells. **(F)** The same cross-section as **(E)** observed by epi-fluorescence showing the blue-violet fluorescence of papillae and two types of fluorescent emission corresponding to the substances accumulated between cortical cells, blue (Blue arrowheads corresponding to lignin) and orange (Red arrowheads). **(G)** TBO-stained longitudinal section of the resistant accession P633 at 4 dpi showing accumulation of phenolic substances (turquoise green staining indicated by arrowheads) at epidermis in presence of fungi. **(H)** TBO-stained cross-section of the resistant accession P633 roots at 4 dpi showing the accumulation of phenolic substances (Blue arrows) between cortical cells. Bar = 25 μm.

TBO staining indicated that the restriction of fungal growth at the outer root area was mainly associated with lignification of host cell walls, detected by their turquoise-green coloration (Baayen et al., 1996), and by the formation of papillae at sites of hyphal penetration in epidermis, exodermis (**Figures 3A,B**) and cortex (**Figures 3C–F**). Marked differences were detected between accessions on the extent of cell wall strengthening and papilla formation in these root tissues (**Figure 2B**). Cell wall reinforcement at the epidermal cell layer (**Figure 3A**) was observed in all accessions although, it was most frequently detected in JI1760 (**Figure 2B**). At exodermis, frequent cell wall reinforcement was observed in all resistant accessions except P633 (**Figure 2B**). In addition, Messire together with JI1412 showed high frequency of cortical cell wall strengthening (**Figures 2B**, **3C,D**).

The formation of papillae to block fungal penetration (**Figures 3A,B**) was much more frequent at the exodermis than at the epidermis and cortical cell layers. At the epidermis, papilla formation was detected only in the resistant accession P42 and to a lesser extent in JI1760 and JI2480 (**Figure 2B**). At the exodermis, papillae were detected in all accessions although they were more frequent in resistant accessions and in the partially resistant accession Messire (**Figure 2B**). By contrast, in cortical cells, papillae (**Figure 3D**) were only frequently observed in the resistant accession JI1412, the partially resistant accessions JI2480 and Messire, and the susceptible accession P629 (**Figure 2B**).

In some accessions including P633, plant defense reaction was also evidenced by the accumulation of a turquoise green substance surrounding the fungus at epidermis (**Figure 3G**) and cortex apoplast (**Figure 3H**). Such staining pattern suggested the accumulation of (poly)phenolic substances within the intercellular spaces (Baayen et al., 1996). AGS staining supported this hypothesis since it revealed a red stained filling substance between cortical cells (**Figure 3E**) showing a blue fluorescence under UV light characteristic of phenolic compounds (**Figure 3F**). This staining also revealed accumulation of other undetermined substances also stained in red but presenting an orange fluorescence under UV light, within intercellular spaces and cell walls of the cortex (**Figure 3F**). Accumulation of (poly)phenolic substances around the fungus in the epidermis (**Figure 3C**) was mainly observed in P633 and to a lesser extent in JI1760 (**Figure 2B**). It was not detected in the rest of the accessions (**Figure 2B**). The accumulation of (poly)phenolic compounds in intercellular spaces of the cortical cell layers was frequently detected in the resistant accession P633 (**Figure 3H**). This host reaction was also detected in P42 and JI1760. By contrast the orange auto-fluorescent filling substances was only detected in the resistant accessions JI1760 and P633 (**Figure 3F**).

#### Endodermis

**FIGURE 4:**
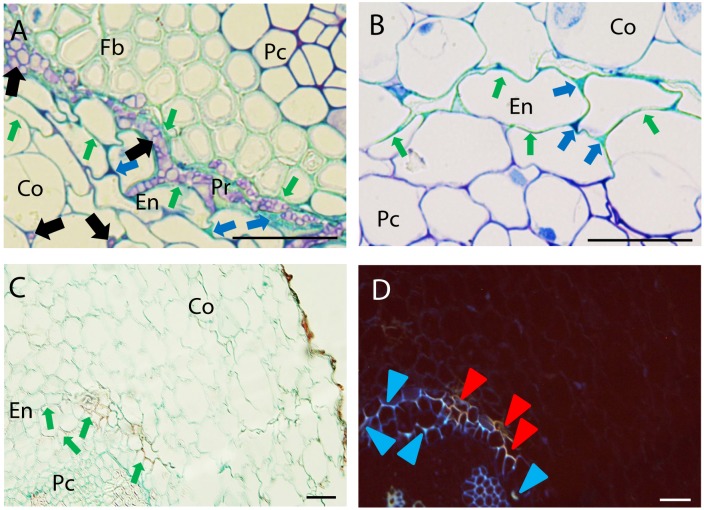
Defense responses and pathogen infection observed at root endodermis in resistant pea accessions after inoculation with *Fop* race 2. **(A)** Cross-section of the partially resistant Messire at 7 days post-inoculation (dpi) stained with TBO showing cell wall strengthening of the endodermal cell walls (Green arrows) accumulation of phenolic and carbohydrates compounds (Blue arrows) stained in turquoise-green and dark purple respectively in the intercellular spaces of endodermis (En) and pericycle (Pr) surrounding fungal cells (Black arrows) Note the presence of fungus in the intercellular spaces of cortical cells and its accumulation between endodermal and pericycle cell layers. **(B)** TBO-stained cross-section of the partially resistant JI2480 at 4 dpi showing the strengthening of endodermal cell walls (turquoise green staining indicated by Green arrows) and the accumulation of phenolics (stained turquoise green) and carbohydrates (dark purple) between endodermal cells (Blue arrows). **(C)** Cross-section of the resistant accession JI1412 at 7 dpi, stained with alcian green:safranin mixture (AGS) showing strengthening of endodermal cell wall (red staining indicated by Green arrows). **(D)** The same cross-section as **(C)** observed under epi-fluorescence showing blue and orange fluorescent emissions of endodermal cell wall suggesting their lignification and suberisation respectively (Blue and Red arrowheads). Bar = 25 μm.

Two main cell reactions were detected in endodermis and pericycle (**Figure 4**). Histological cross section of resistant and partially resistant accessions evidenced an intense cell wall lignification and suberisation of endodermal cells as revealed by their characteristic turquoise green coloration after TBO staining (**Figures 4A,B**) and their red coloration after AGS staining fluorescing blue or orange under UV excitation (**Figures 4C,D**). This cell response was frequently detected in the resistant accessions P42 and JI1412 and in the partially resistant accessions Messire and JI2480 while it was only rarely detected in the other accessions (**Figure 2B**).

Concomitantly, accumulation of (poly)phenolic compounds (stained turquoise green by TBO and red by AGS; **Figures 4A,C**) and carbohydrates (stained in dark purple by TBO; **Figures 4A,B**) was observed in the intercellular space of endodermis. Accumulation of (poly)phenolic compounds in the intercellular spaces of endodermis was frequently detected in the resistant accession P42 and in the partially resistant accessions Messire and JI2480 (**Figure 2B**). In turn, carbohydrates accumulation around pericycle was frequently detected in all accessions except in the resistant accession JI1760 and the partially resistant accession JI2480 (**Figure 2B**).

The intense cell wall strengthening and accumulation of phenolic and carbohydrates compounds at endodermis and pericycle might create a physical and chemical barrier halting the fungal progression toward vascular stele as exemplified by the intense fungal colonization of intercellular space just beneath endodermis in Messire (**Figure 4A**).

#### Stele

**FIGURE 5:**
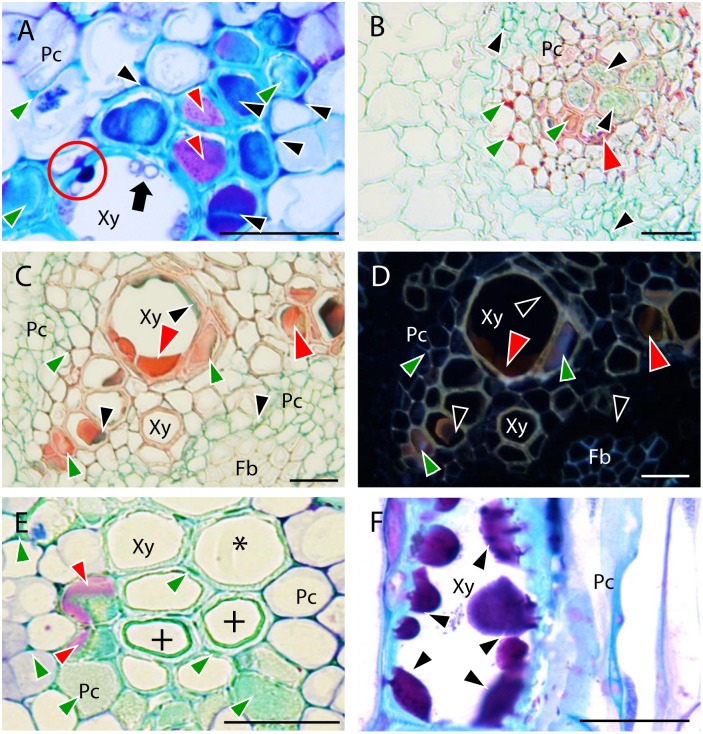
Defense responses observed in root vascular tissues of resistant pea accessions after inoculation with *Fop* race 2. Black arrows indicate fungus. **(A)** Cross-section of the resistant accession JI1412 at 7 dpi stained with TBO showing carbohydrate accumulation (dark purple staining indicated by Black arrowheads) between vascular cells and entering infected xylem cells (red circle) to trap fungal cells (Black arrows). The turquoise blue coloration accumulating within and between completely plugged vascular cells (Green arrowheads) corresponds to polyphenols and the clear purple staining (Red arrowheads) to other filling substances. **(B)** Cross-section of the resistant accession P633 at 7 dpi stained with AGS showing accumulation of polyphenols (red staining indicated by Green arrowheads), carbohydrates (green staining indicated by Black arrowheads) and other filling substances (dark red staining indicated by Red arrowheads) within and between vascular cells. **(C)** Cross-section of the resistant P42 roots at 7 dpi stained with AGS showing accumulation of polyphenols (red staining indicated by Green arrowheads) and carbohydrates (green staining indicated by Black arrowheads) and other filling substances (dark red staining indicated by Red arrowheads) within and between vascular cells. **(D)** The same as **(C)** observed under epi-fluorescence showing the fluorescent emission of polyphenols (Green arrowheads). Black arrowheads indicate carbohydrates and Red arrowheads indicate other non-fluorescent filling substances. **(E)** TBO-stained cross-section of the resistant accession JI1412 roots at 7 dpi showing the cell wall strengthening of xylem cells (+). Asterisk indicates a normal vessel as a reference. Note also the accumulation of phenolics between xylem parenchyma cells and in the lumen of some xylem cells (turquoise green staining, Green arrowheads) and accumulation of other filling substance in some of these xylem cells (clear purple staining, Red arrowheads). **(F)** TBO-stained longitudinal section of the resistant accession JI1412 at 7 dpi showing the filling of a xylem vessel by carbohydrates originating from intercellular vascular cells progressively plugging the xylem vessel (Black arrowheads). Bar = 25 μm.

Intense cell responses were detected in the stele of resistant and partially resistant accessions at 4 and 7 dpi following root trimming inoculation. By contrast they were only detected in partially resistant accessions following *Fop* inoculation without root trimming. Stele colonization by *Fop* induced three main cell reactions in resistant and partially resistant accessions including (i) clogging of xylem vessels, (ii) accumulation of phenolic compounds and carbohydrates in intercellular spaces surrounding colonized vessels and (iii) vessel wall thickening (**Figure 5**). At least three different compounds were detected within xylem vessels and surrounding intercellular spaces. Staining with TBO and AGS indicated that the main substances were mucilage, a polysaccharide, evidenced by its dark purple coloration after TBO staining (**Figure 5A**; Western et al., 2000) and non-fluorescent green coloration after AGS staining (**Figures 5B–D**) and (poly)phenolic compounds including lignin stained turquoise green by TBO (**Figures 5A,E**) and red with blue fluorescence by AGS (**Figures 5B–D**). Another undetermined filling substance stained clear purple by TBO (**Figures 5A,E**) and non-fluorescing dark red by AGS (**Figures 5C,D**) was also often detected in xylem cells.

All resistant and partially resistant accessions, except JI1760, showed very frequent (scoring value of 4) accumulation of polyphenols (**Figures 5B–D**) in the intercellular spaces of the stele (**Figure 2B**). At this site, accumulation of carbohydrates was also very frequently detected in the resistant accessions JI1412, P633 and P42 (**Figures 5A,B**), whereas it was rarely detected in the other accessions (**Figure 2B**). Within xylem vessels, accumulation of polyphenols was more frequent in the resistant accessions P42 and JI1412 (**Figure 2B**) in which some proto-xylem cells were completely clogged by polyphenols (**Figures 5A,E**). The other resistant accessions (P633 and JI1760) and the partially resistant accession JI2480 also significantly accumulated polyphenols within xylem vessels, whereas it was rarely detected in the partially resistant accession Messire and in the susceptible accessions (**Figure 2B**). Accumulation of carbohydrates was detected in xylem vessels of all accessions. However, this accumulation was only frequently observed in the resistant accessions P633, JI1412 and P42 (**Figure 2B**). A detailed microscopic observation showed that the mucilages clogging xylem vessels originated from the intercellular spaces surrounding affected xylem vessels and entered them through the pits until complete cell filling (**Figures 5A,F**). Accumulation of the other occluding material stained in clear purple by TBO, was also observed in xylem vessels of most accessions except the susceptible accession P629 and the partially resistant accession JI2480 (**Figure 2B**).

In addition to the accumulation of these compounds, the thickening of vessel cell walls was also detected in some resistant accessions (**Figure 5E**). This reaction was frequently detected in the resistant accession JI1412 and to a lesser extent in the resistant accession P42 (**Figure 2B**). The turquoise-green appearance of vessel thickenings after TBO staining (**Figure 5E**) suggested that it was formed by deposition of (poly)phenolic materials more likely to be lignin.

### Relative Contribution of Specific Root Tissue and Cell Defense Reaction in Pea Resistance to *Fop*

**FIGURE 6:**
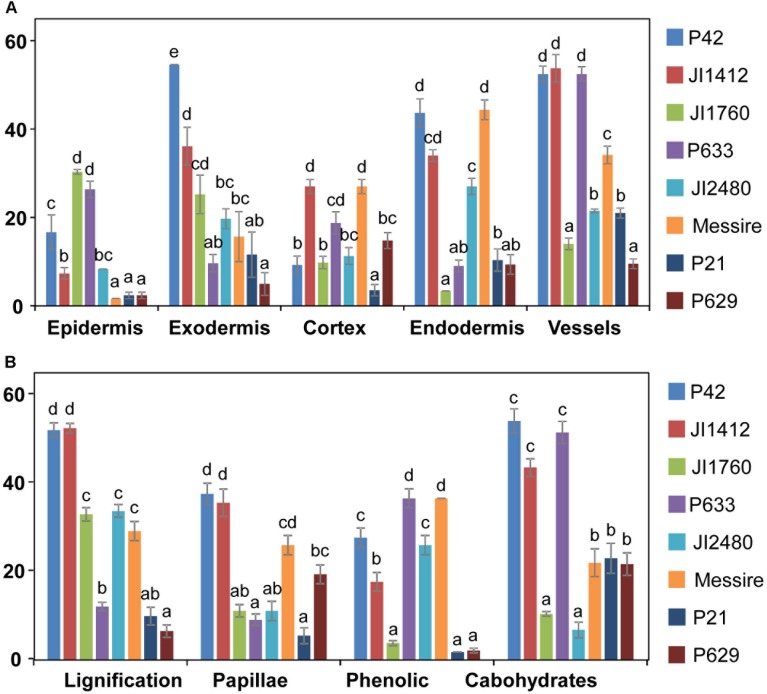
Relevance of tissue and resistance responses in the defense strategy of pea against *F. oxysporum*. Data compare the frequency of overall resistance responses at each root tissue **(A)** and frequency of each defense reaction **(B)** between pea accessions with different levels of resistance to *F. oxysporum*. Vertical bars are standard errors for *n* = 10. Different letters indicate significant differences at *p* < 0.05 according to Tukey’s range test.

To determine the relative contribution of each root tissue and defense mechanism in *Fop* resistance, we compared the frequency of defense reactions between pea accessions according to root tissue (**Figure 6A**) or defense mechanism (**Figure 6B**). Significant differences between accessions were detected for both the localization and type of defense mechanism suggesting that each resistant accession had developed its own defense strategy. Analyses of variances confirmed these results since they revealed not only significant differences between accessions, root tissues and defense mechanisms (*p <* 0.001) but also significant interactions between these factors (*p* < 0.001).

**FIGURE 7:**
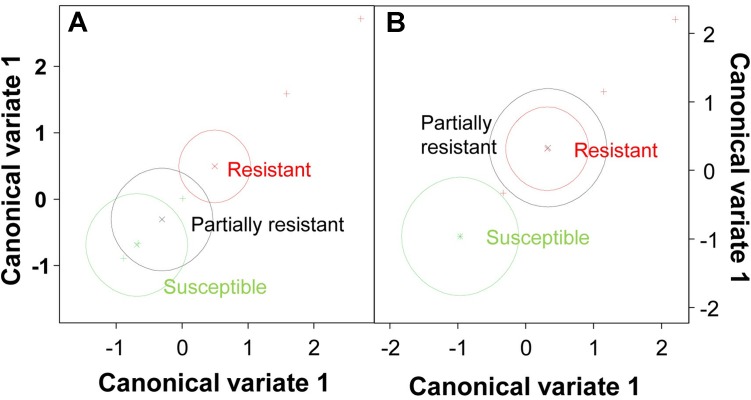
Multivariate analysis of pea genotypes. Scatterplot of canonical variate analysis scores of components 1 and 2 based on the frequency of resistance responses observed at epidermis/exodermis **(A)** and lignification **(B)**.

To identify the key elements of pea defense to *Fop*, correlation analysis between the measured cell responses and disease severity was performed (**Tables 2**, **3**). Interestingly, this analysis revealed high negative correlation between the response of epidermal and exodermal cells and disease severity (*r* = -0.83, *p* < 0.01 and *r* = -0.67, *p* < 0.05 respectively). Although high correlation with disease severity was also detected in other root tissues including xylem vessels, they were not significant (**Table 2**). Among the cell defense reactions, only lignification was significantly and negatively correlated with disease severity (*r* = -0.64, *p* < 0.05) (**Table 3**). This suggests a key role of the outer root region and of lignification in pea resistance to *Fop.* Canonical variate analyses confirmed these results since the responses engaged at the outer root region (epidermis and exodermis) or lignification allowed discriminating between susceptible and resistant accessions (**Figure 7**).

**Table 2:** Pearson correlations between defense response establishment at different tissues (cortex, epidermis, endodermis, exodermis, and vessels) and disease severity.

	Severity	Cortex	Endodermis	Epidermis	Exodermis	Vessels
Severity						
Cortex	-0.2063					
Endodermis	-0.1930	0.4420				
Epidermis	-0.8336**	-0.0790	-0.3327			
Exodermis	-0.6703*	-0.0327	0.6109	0.2854		
Vessels	-0.6301	0.4578	0.5046	0.2583	0.5731	


*^∗^, ^∗∗^, ^∗∗∗^Indicate significance at *p* < 0.05, 0.01, and 0.001, respectively.*

**Table 3:** Pearson correlations between particular defense responses (cell wall lignification, papillae formation, accumulation of phenolics and carbohydrates) and disease severity.

	Severity	Lignification	Papillae	Phenolics	Carbohydrates
Severity					
Lignification	-0.6498^∗^				
Papillae	-0.3749	0.6695^∗^			
Phenolics	-0.5963	0.5315	0.5257		
Carbohydrates	-0.4758	0.1109	0.5214	0.6687^∗^	


*^∗^, ^∗∗^, ^∗∗∗^Indicate significance at *p* < 0.05, 0.01, and 0.001, respectively.*

## Discussion

*Fop* is one of the major constraints of pea worldwide. Defining the defense mechanisms acting in sources of resistance is crucial for a more targeted and efficient breeding. Reaching a deep understanding of *Fop* infection process is also important to define efficient control strategies. Thus, we performed a detailed histological study on eight pea accessions with differential responses to *Fop* race 2 isolates (Bani et al., 2012). Although several histological studies has characterized the mechanisms of resistance to *F. oxysporum* in several plant species including pea (Bishop and Cooper, 1984; Baayen et al., 1989; Tessier et al., 1990; Shi et al., 1992; Ouellette et al., 1999; Zvirin et al., 2010; Pereira et al., 2013), our study is the first comparative histological study on several resistant accessions. This revealed a large variety of responses taking place throughout the fungal colonization sites from root epidermis to vascular stele and indicated the importance of the outer root region and of lignification in *Fop* resistance.

Successful host infection by *F. oxysporum* is a complex process that can be divided in four main steps, (1) recognition of host root, (2) attachment and superficial colonization of host root, (3) penetration of root epidermis and progression through cortex and (4) colonization of xylem vessel. Although the histology of *F. oxysporum* infection has been studied in details by light, confocal and electron microscopy, several steps of this process remain under debate including how *F. oxysporum* penetrates host root, colonizes cortical cells and spreads within xylem vessels. Our observation in pea may contribute to clarify some of these steps.

It is generally accepted that *F. oxysporum* penetrates root directly without the formation of specialized appresorium-like structures (Bishop and Cooper, 1983b). Several reports further evidenced that epidermal cell wall was breached by the differentiation of constricted hyphae or infective hyphae that recover their normal size upon cell wall penetration (Bishop and Cooper, 1983a; Rodríguez-Gálvez and Mendgen, 1995). By contrast, several recent studies in *Arabidopsis* and watermelon observed the swelling of infective hyphae forming rudimentary appressorium-like penetration structures (Czymmek et al., 2007; Lü et al., 2014). In pea, our observations suggested the direct penetration of host root (**Figure 1B**). Swelling of infective hypha before infection was not observed in our root preparations. Instead, the constriction of the penetrating hypha at the site of cell penetration was recurrently observed at the epidermis, exodermis and in the cortex (**Figures 1B,D**) supporting the process of penetration previously described by electron microscopy in tomato, pea and cotton (Bishop and Cooper, 1983a; Rodríguez-Gálvez and Mendgen, 1995; Benhamou and Garand, 2001).

Once inside the root, most studies described the intercellular progression of *F. oxysporum* through the cortex (Kroes et al., 1998; Jiménez-Fernández et al., 2013; Niño-Sánchez et al., 2015). This exclusively apoplastic progression was also reported in pea following root dipping inoculation (Bishop and Cooper, 1983a). In this species, the intracellular progression of *Fop* through cortex was also reported following inoculation of *Ri*-transformed pea root culture (Benhamou and Garand, 2001). Here, following the root dipping inoculation, an abundant colonization of cortical cells by *Fop* was detected in susceptible accessions. As reported in **Figure 1D**, numerous infective hyphae were observed moving in and out of cortical cells. In most cases cell-to-cell movement was mediated by constricted hypha as previously reported (**Figure 1D**; Benhamou and Garand, 2001). Recent studies in cabbage and lentil also reported the intracellular colonization of cortical cells (Li et al., 2015; Pouralibaba et al., 2017). Intracellular progression of *F. oxysporum* through the cortex may thus be more common than previously though. Interestingly, *Fop* progression in root of resistant and partially resistant pea accessions was strictly intercellular due to the establishment of efficient defense reactions (**Figure 1E**). This indicates that plant genotype is an important determinant of *F. oxysporum* progression. Therefore the difference in root cortex colonization might reflect differences in the susceptibility of the pea accessions used. Colonization of susceptible accessions was also associated with the degradation of the cell layers surrounding endodermis (**Figures 1**, **6A,B**) as previously shown for several *formae speciales* of *F. oxysporum* (Tessier et al., 1990; Kroes et al., 1998; Olivain and Alabouvette, 1999; Ouellette et al., 1999; Benhamou and Garand, 2001). Cell wall degrading enzymes (CWDE) have been recently identified as key pathogenicity factors that contribute to successful *F. oxysporum* infection in tomato (Bravo Ruiz et al., 2016). The intensive cell degradation detected here suggested that CWDE might also be involved in the pathogenesis of *Fop* race 2.

The last steps of *F. oxysporum* infection are the colonization of xylem vessels and its subsequent spreading to the hypocotyl. Traditionally, it is though that *F. oxysporum* infect xylem vessel through the pits or by breaching cell walls of immature xylem elements (Bishop and Cooper, 1983a,b). In susceptible pea accession, *Fop* was detected entering immature and mature xylem vessel alike by direct penetration of xylem cell wall. Abundant inter- and intra-cellular colonization of fiber and parenchyma cells was also often observed in these pea accessions (**Figure 1F**). By contrast, penetration of mature xylem vessel was not observed in resistant and partially resistant accessions. Nevertheless, the restricted presence of *Fop* cells in xylem vessels in partially resistant accessions inoculated without root trimming indicates that in these accessions the fungus can overcome plant defense by infecting immature vascular tissue. Altogether these observations also support that *Fop* infection process depends at least partly on the level of susceptibility of its host. Once the *F. oxysporum* enter within xylem vessels, it colonizes them by growing longitudinally in both directions. According to literature, intra-tracheary colonization is either accomplished by germination of microconidia through xylem perforation plates (Beckman, 1987) or by hypha elongation of the growing *F. oxysporum* colonies (Czymmek et al., 2007; Michielse and Rep, 2009). Observation of numerous longitudinal sections of inoculated pea roots revealed that intra-tracheary movement was made mainly by elongation of infective hypha (**Figure 1G**) although germination of microconidia through xylem perforation plates was also detected in a few instances (**Figure 1H**). Our observations also showed that the fungus spread in vascular tissue by colonizing adjacent vessels through elongation of infective hypha breaching their cell walls (**Figure 1G**). These observations would support the minor role of microconidia in the colonization of xylem vessels and hypocotyl previously reported (Czymmek et al., 2007; Michielse and Rep, 2009).

Resistance to fusarium wilt has been associated to several defensive mechanisms. Three main areas were differentiated, the outer root section, the endodermis and the vascular stele. Previous studies in flax, tomato and watermelon demonstrated the key role of the outer root region (epidermis, exodermis and cortex) in the resistance (Kroes et al., 1998; Olivain and Alabouvette, 1999; Olivain et al., 2003; Lü et al., 2014). Other studies, performed in pea or carnation showed that the main discriminating factor between susceptibility and resistance localized at the endodermis (Bishop and Cooper, 1983b; Baayen et al., 1989). Here, we observed the coordinated establishment of different mechanisms across pea root tissues to efficiently block the pathogen progression before vascular stele. Significant differences in the intensity of defense reaction at each root area were detected between accessions (**Figure 6A**). This suggests that the contribution of each root area to resistance depends on the accessions. Despite these differences, the outer root region, including epidermis and exodermis, was identified as the major determinant of resistance since defense reactions established at this region was highly correlated with disease severity and their frequency discriminates between resistant and susceptible accessions (**Figure 7A** and **Table 2**). Interestingly, this region did not discriminate partially resistant from susceptible accessions (**Figure 7A**) suggesting that the main discriminating defense element might take place in another location in these accessions. Strong disease reaction at endodermis was detected in both partially resistant accessions tested (**Figures 2B**, **6A**) that were also characterized by the massive accumulation of fungal cell at this level (**Figure 4A**). Endodermis would thus appear as the main discriminant factor of partially resistant accessions while it only plays a minor role in resistant accessions since fungal progression was most often blocked before this region. Despite the absence of fungus in this area, endodermis strengthening may have a secondary role in the resistant accessions by impeding the fungus growing out of infected xylem vessels.

A battery of cell defense reactions have been previously related to *F. oxysorum* resistance including, cell wall strengthening, papilla formation, production of antifungal compounds, accumulation of gums, gels or tyloses within xylem cells and vessel crushing (Bishop and Cooper, 1983a; Baayen and Elgersma, 1985; Beckman, 1987; Charchar and Kraft, 1989; Ouellette et al., 1999; Grayer and Kokubun, 2001; Yadeta and Thomma, 2013). The importance of each of these cell reactions in resistance remains unclear. Our thorough histological analysis revealed that resistance was mainly based on three defense mechanisms, papilla formation, cell wall strengthening and accumulation of (poly)phenolics and carbohydrates. Although all accessions combined these mechanisms, differences in the frequency of these defensive reactions, were detected between accessions (**Figures 2B**, **6B**) suggesting that each accessions develop its own defense strategy. Despite these variations, cell wall strengthening mechanisms through lignification was identified as the main determinant of resistance. Indeed, lignification was the unique defense mechanism significantly correlated with resistance and the sole mechanism discriminating between (partially) resistant and susceptible accessions (**Figure 7B**). Lignification might contribute to resistance through different actions. Lignification can not only strengthen cell wall making them more resistant to the compressive forces exerted by fungal pathogen during infection attempts but it might also shield cell wall components preventing their degradation (Vance et al., 1980; Lattanzio et al., 2006). The intense cell wall lignification detected here might thus form a physical barrier to the pathogen entry. Lignification has also been postulated to restrict diffusion of fungal secreted enzymes or toxins. In addition, lignins and its derivatives can bind and inhibit CWDE (Walters et al., 2007; Vidhyasekaran, 2008). Thus, the intense lignification and suberisation of cortical and endodermal cell wall detected in resistant pea accessions (**Figures 3**, **4**) might contribute to neutralize fungal CWDE.

In addition to the extensive lignification of cell walls of resistant accessions, we observed formation of papillae at sites of attempted hyphae penetration at the exodermis and cortex. This localized cell wall strengthening mechanism was particularly effective in some resistant accessions such as JI1412 and P42 impeding pathogen entry within cell and its intracellular progression through root. Accumulation of (poly)phenolics and carbohydrates was also detected at epidermis, exodermis, cortex, endodermis and the vascular stele forming five spatially differentiated chemical barriers within the roots. Altogether, these different mechanisms contributed to create physical and chemical barriers that efficiently blocked and killed the invading pathogen before reaching the stele.

Several mechanisms have been shown to limit fungal growth within xylem vessel and to block its vertical spread in several species including pea (Beckman et al., 1972; Baayen et al., 1989; Tessier et al., 1990; Shi et al., 1992; Zvirin et al., 2010; Pereira et al., 2013). In order to detect these defense mechanisms, we performed a series of inoculation experiments with root trimming that favor direct fungal entry in the vascular tissues. In this way, most of these mechanisms could be detected in resistant accessions that otherwise would had completely blocked fungal invasion at outer cell layers. These mechanisms were also detected in partially resistant accessions independently to the inoculation method. These mechanisms consisted mainly in vessel clogging or occlusion with materials, usually denoted as gels, gums or mucilages. Mucilage production has been previously reported as a defensive response against the parasitic plant *Orobanche crenata* (Pérez-De-Luque et al., 2005, 2006) indicating that it might be a general mechanism to prevent spreading of invading organisms through xylem. The main mucilage components are carbohydrates (specifically pectins) and polyphenols although they can also contain phytoalexins, lignin-like compounds or lipoidal substances (Niemann et al., 1990; Shi et al., 1992; Baayen et al., 1996; Pérez-De-Luque et al., 2006; Hall et al., 2011). Upon conducive conditions, we observed the important production of carbohydrates, (poly)phenolics and additional undetermined substances by vascular parenchyma cells and their progressive accumulation in the lumen of xylem cells, which supports the origin and composition of mucilage previously reported. Our data shows that accumulation of phenolic compounds and carbohydrates was higly correlated, supporting the relevance of both sustances as resistance response at xylem vessels. Although the nature of the additional occluding substances are unknown, their staining pattern and mode of accumulation suggest that they might be formed by other carbohydrates and/or lipidic substances known to be also present in mucilages (Niemann et al., 1990; Shi et al., 1992). Apart from mucilage, we detected the thickening of vessel cell walls controlling lateral spreading of the pathogen. Vessel wall thickening has been reported as a typical defense reaction against *F. oxysporum* in many species (Tessier et al., 1990; Shi et al., 1992; Baayen et al., 1996; Ouellette et al., 2002; Pereira et al., 2013). The phenolic and lignin nature of these coatings may contribute to chemical inhibition and physical restriction of the fungus within vessels (Shi et al., 1992; Hilaire et al., 2001).

Our observation supports a key function of phenolic compounds in pea defense against *Fop*, although further studies would be needed to identify the phenolic compounds accumulating in the resistant pea roots and confirm their role. Beside the intense lignification that might contribute to strengthen cell walls and neutralize CWDE action, our study revealed the frequent accumulation of (poly)phenolic compounds both inter and intracellular near fungal cells (**Figures 3**–**5**). Phytoalexins such as pisatin and lignans are well-known defense metabolites due to their potent anti-fungal activities and their capacity to inhibit secreted fungal enzymes (Lattanzio et al., 2006). Accumulation of (poly)phenolics in response to *Fop* was previously evidenced. A recent proteomic analysis of the resistant pea-*Fop* interaction revealed the accumulation of several enzymes of the phenylpropanoid pathways involved in the synthesis of (poly)phenolic compounds (Castillejo et al., 2015). In addition, *Fop* inoculation was found to induce pisatin accumulation in resistant pea roots suggesting a role of pisatin in quantitative resistance in pea against *Fop* (Bani et al., 2018). Although their function is still unclear, our results further supports the important role of (poly)phenolic compounds in *Fop* resistance.

Altogether, our results clarified the infection process of *Fop* in susceptible and resistant pea accessions. More importantly, our results indicated that resistant accessions developed different mechanisms creating physical and chemical barriers to impede the pathogen progression toward the vascular stele. Interestingly, it showed quantitative rather than qualitative differences between accessions that differed between each other in the frequency and the spatial distribution of the established defense reactions. This study contributes to our understanding of the resistance mechanisms that efficiently block *F. oxysporum* infection and it advances our understanding of the mechanisms acting in pea against *Fop* race *2*. Potentiating the different defense mechanisms detected in these pea accessions in a single resistant cultivar through breeding would contribute to control this important pea disease and improve the durability of existing resistance levels in the field.

## Author Contributions

MB, NR, and DR conceived and designed the experiments. MB performed the experiments. MB, NR, and AP-D-L discussed and interpreted the data. MB and NR drafted and wrote the manuscript. AP-D-L and DR also participated in the critical reading, writing, and correction of the manuscript.

## Conflict of Interest Statement

The authors declare that the research was conducted in the absence of any commercial or financial relationships that could be construed as a potential conflict of interest.

## References

[B1] Baayen R. P. Elgersma D. M. (1985). Colonization and histopathology of susceptible and resistant carnation cultivars infected with *Fusarium oxysporum* f.sp. *dianthi.* *Neth. J. Plant Pathol.* 91 119–135. 10.1007/bf01976386

[B2] Baayen R. P. Ouellette G. B. Rioux D. (1996). Compartmentalization of decay in carnations resistant to *Fusarium oxysporum* f. sp. *dianthi*. *Phytopathology* 86 1018–1031. 10.1094/Phyto-86-1018

[B3] Baayen R. P. Vaneijk C. Elgersma D. M. (1989). Histology of roots of resistant and susceptible carnation cultivars from soil infested with *Fusarium oxysporum* f. sp. *dianthi*. *Neth. J. Plant Pathol.* 95 3–13. 10.1007/bf02000875

[B4] Baird D. B. Harding S. A. Lane P. W. Murray D. A. Payne R. W. Soutar D. M. (2002). *Genstat for Windows (6th Edition) Introduction.* Oxford: VSN International.

[B5] Bani M. Cimmino A. Evidente A. Rubiales D. Rispail N. (2018). Pisatin involvement in the variation of inhibition of *Fusarium oxysporum* f. sp. *pisi* spore germination by root exudates of *Pisum* spp. germplasm. *Plant Pathol.* (in press) 10.1111/ppa.12813

[B6] Bani M. Rubiales D. Rispail N. (2012). A detailed evaluation method to identify sources of quantitative resistance to *Fusarium oxysporum* f. sp. *pisi* race 2 within a *Pisum* spp. germplasm collection. *Plant Pathol.* 61 532–542. 10.1111/j.1365-3059.2011.02537.x

[B7] Beckman C. H. (1987). *The Nature of Wilt Diseases of Plants.* St Paul, MN: APS Press.

[B8] Beckman C. H. Elgersma D. M. Machardy W. E. (1972). Localization of fusarial infections in vascular tissue of single-dominant-gene resistant tomatoes. *Phytopathology* 62 1256–1260. 10.1094/Phyto-62-1256

[B9] Benhamou N. Garand C. (2001). Cytological analysis of defense-related mechanisms induced in pea root tissues in response to colonization by nonpathogenic *Fusarium oxysporum* Fo47. *Phytopathology* 91 730–740. 10.1094/phyto.2001.91.8.730 18944029

[B10] Bishop C. D. Cooper R. M. (1983a). An ultrastructural study of root invasion in 3 vascular wilt diseases. *Physiol. Plant Pathol.* 22 15–27. 10.1016/S0048-4059(83)81034-0

[B11] Bishop C. D. Cooper R. M. (1983b). An ultrastructural study of vascular colonization in 3 vascular wilt diseases. 1. Colonization of susceptible cultivars. *Physiol. Plant Pathol.* 23 323–343. 10.1016/0048-4059(83)90018-8

[B12] Bishop C. D. Cooper R. M. (1984). Ultrastructure of vascular colonization by fungal wilt pathogens.2. Invasion of resistant cultivars. *Physiol. Plant Pathol.* 24 277–289. 10.1016/0048-4059(84)90002-x

[B13] Bravo Ruiz G. Di Pietro A. Roncero M. I. G. (2016). Combined action of the major secreted exo- and endopolygalacturonases is required for full virulence of *Fusarium oxysporum*. *Mol. Plant Pathol.* 17 339–353. 10.1111/mpp.12283 26060046 PMC6638378

[B14] Castillejo M. A. Bani M. Rubiales D. (2015). Understanding pea resistance mechanisms in response to *Fusarium oxysporum* through proteomic analysis. *Phytochemistry* 115 44–58. 10.1016/j.phytochem.2015.01.009 25672548

[B15] Charchar M. Kraft J. M. (1989). Response of near-isogenic pea cultivars to infection by *Fusarium oxysporum* f. sp. *pisi* races 1 and 5. *Can. J. Plant Sci.* 69 1335–1346. 10.4141/cjps89-161

[B16] Ciancio A. Mukerji K. G. (2008). *Integrated Management of Diseases Caused by Fungi, Phytoplasma and Bacteria.* Dordretch: Springer 10.1007/978-1-4020-8571-0

[B17] Crews L. J. McCully M. E. Canny M. J. (2003). Mucilage production by wounded xylem tissue of maize roots - time course and stimulus. *Funct. Plant Biol.* 30 755–766. 10.1071/fp03052 32689059

[B18] Czymmek K. J. Fogg M. Powell D. H. Sweigard J. Park S. Y. Kang S. (2007). In vivo time-lapse documentation using confocal and multi-photon microscopy reveals the mechanisms of invasion into the *Arabidopsis* root vascular system by *Fusarium oxysporum*. *Fungal Genet. Biol.* 44 1011–1023. 10.1016/j.fgb.2007.01.012 17379550

[B19] Grayer R. J. Kokubun T. (2001). Plant-fungal interactions: the search for phytoalexins and other antifungal compounds from higher plants. *Phytochemistry* 56 253–263. 10.1016/s0031-9422(00)00450-7 11243452

[B20] Hall C. Heath R. Guest D. I. (2011). Rapid and intense accumulation of terpenoid phytoalexins in infected xylem tissues of cotton (*Gossypium hirsutum*) resistant to *Fusarium oxysporum* f. sp. *vasinfectum*. *Physiol. Mol. Plant Pathol.* 76 182–188. 10.1016/j.pmpp.2011.09.002

[B21] Hilaire E. Young S. A. Willard L. H. McGee J. D. Sweat T. Chittoor J. M. (2001). Vascular defense responses in rice: peroxidase accumulation in xylem parenchyma cells and xylem wall thickening. *Mol. Plant Microbe Interact.* 14 1411–1419. 10.1094/mpmi.2001.14.12.1411 11768536

[B22] Infantino A. Kharrat M. Riccioni L. Coyne C. J. McPhee K. E. Grunwald N. J. (2006). Screening techniques and sources of resistance to root diseases in cool season food legumes. *Euphytica* 147 201–221. 10.1007/s10681-006-6963-z

[B23] Jiménez-Fernández D. Landa B. B. Kang S. Jiménez-Díaz R. M. Navas-Cortés J. A. (2013). Quantitative and microscopic assessment of compatible and incompatible interactions between chickpea cultivars and *Fusarium oxysporum* f. sp. *ciceris* races. *PLoS One* 8:e61360. 10.1371/journal.pone.0061360 23613839 PMC3629054

[B24] Joel D. M. (1983). AGS (alcian green safranin): a simple differential staining of plant material for the light microscope. *Proc. R. Microsc. Soc.* 18 149–151.

[B25] Karnovsky M. J. (1965). A formaldehyde-glutaraldehyde fixative of high osmolality for use in electron microscopy. *J. Cell Biol.* 27 137A–138A.

[B26] Kraft J. M. Haware M. P. Jiménez-Díaz R. M. Bayaa B. Harrabi M. (1994). Screening techniques and sources of resistance to root rots and wilts in cool-season food legumes. *Euphytica* 73 27–39. 10.1007/bf00027179

[B27] Kroes G. M. L. W. Baayen R. P. Lange W. (1998). Histology of root rot of flax seedlings (*Linum usitatissimum*) infected by *Fusarium oxysporum* f. sp. *lini*. *Eur. J. Plant Pathol.* 104 725–736. 10.1023/a:1008604417614

[B28] Lattanzio V. Lattanzio V. M. T. Cardinali A. (2006). “Role of phenolics in the resistance mechanisms of plants against fungal pathogens and insects,” in *Phytochemistry: Advances in Research*, ed. Imperato F. (Kerala: Research Signpost), 23–67.

[B29] Li E. Wang G. Yang Y. Xiao J. Mao Z. Xie B. (2015). Microscopic analysis of the compatible and incompatible interactions between *Fusarium oxysporum* f. sp. *conglutinans* and cabbage. *Eur. J. Plant. Pathol.* 141 597–609. 10.1007/s10658-014-0567-6

[B30] Lü G. Guo S. Zhang H. Geng L. Martyn R. D. Xu Y. (2014). Colonization of fusarium wilt-resistant and susceptible watermelon roots by a green-fluorescent-protein-tagged isolate of *Fusarium oxysporum* f.sp. *niveum*. *J. Phytopathol.* 162 228–237. 10.1111/jph.12174

[B31] McPhee K. E. Inglis D. A. Gundersen B. Coyne C. J. (2012). Mapping QTL for fusarium wilt race 2 partial resistance in pea (*Pisum sativum*). *Plant Breed.* 131 300–306. 10.1111/j.1439-0523.2011.01938.x

[B32] Michielse C. B. Rep M. (2009). Pathogen profile update: *Fusarium oxysporum*. *Mol. Plant Pathol.* 10 311–324. 10.1111/j.1364-3703.2009.00538.x 19400835 PMC6640313

[B33] Nelson E. B. (1991). “Exudate molecules initiating fungal response to seed and roots,” in *The Rhizosphere and Plant Growth*, eds Keister D. L. Cregan P. B. (Dortrecht: Kluwer), 197–209.

[B34] Nelson P. E. (1981). “Life cycle and epidemiology of *Fusarium oxysporum*,” in *Fungal Wilt Diseases of Plants*, eds Mace M. E. Bell A. A. Beckman C. H. (New York, NY: Academic Press), 51–80. 10.1016/B978-0-12-464450-2.50008-5

[B35] Niemann G. J. Baayen R. P. Boon J. J. (1990). Localization of phytoalexin accumulation and determination of changes in lignin and carbohydrate-composition in carnation (*Dianthus caryophyllus* L) xylem as a consequence of infection with *Fusarium oxysporum* f. sp. *dianthi*, by pyrolysis mass-spectrometry. *Neth. J. Plant Pathol.* 96 133–153. 10.1007/bf01974252

[B36] Niño-Sánchez J. Tello V. Casado-Del-Castillo V. Thon M. R. Benito E. P. Díaz-Mínguez J. M. (2015). Gene expression patterns and dynamics of the colonization of common bean (*Phaseolus vulgaris* L.) by highly virulent and weakly virulent strains of *Fusarium oxysporum*. *Front. Microbiol.* 6:234. 10.3389/fmicb.2015.00234 25883592 PMC4383042

[B37] Olivain C. Alabouvette C. (1999). Process of tomato root colonization by a pathogenic strain of *Fusarium oxysporum* f. sp. *lycopersici* in comparison with a non-pathogenic strain. *New Phytol.* 141 497–510. 10.1046/j.1469-8137.1999.00365.x 33863075

[B38] Olivain C. Trouvelot S. Binet M. N. Cordier C. Pugin A. Alabouvette C. (2003). Colonization of flax roots and early physiological responses of flax cells inoculated with pathogenic and nonpathogenic strains of *Fusarium oxysporum*. *Appl. Environ. Microbiol.* 69 5453–5462. 10.1128/aem.69.9.5453-5462.2003 12957934 PMC194917

[B39] Ouellette G. B. Baayen R. P. Simard M. Rioux D. (1999). Ultrastructural and cytochemical study of colonization of xylem vessel elements of susceptible and resistant *Dianthus caryophyllus* by *Fusarium oxysporum* f. sp.*dianthi*. *Can. J. Bot.* 77 644–663. 10.1139/b99-033

[B40] Ouellette G. B. Baayen R. P. Simard M. Rioux D. (2002). Reactions of paratracheal cells of resistant and susceptible carnation (*Dianthus caryophyllus*) cultivars to vascular invasion by *Fusarium oxysporum* f. sp. *dianthi*. *New Phytol.* 156 113–128. 10.1046/j.1469-8137.2002.00499.x

[B41] Pereira A. C. Cruz M. F. A. Paula Junior T. J. Rodrigues F. A. Carneiro J. E. S. Vieira R. F. (2013). Infection process of *Fusarium oxysporum* f. sp *phaseoli* on resistant, intermediate and susceptible bean cultivars. *Trop. Plant Pathol.* 38 323–328. 10.1590/S1982-56762013005000022

[B42] Pérez-De-Luque A. Jorrin J. Cubero J. I. Rubiales D. (2005). *Orobanche crenata* resistance and avoidance in pea (*Pisum* spp.) operate at different developmental stages of the parasite. *Weed Res.* 45 379–387. 10.1111/j.1365-3180.2005.00464.x

[B43] Pérez-De-Luque A. Lozano M. D. Cubero J. I. González-Melendi P. Risueño M. C. Rubiales D. (2006). Mucilage production during the incompatible interaction between *Orobanche crenata* and *Vicia sativa*. *J. Exp. Bot.* 57 931–942. 10.1093/jxb/erj078 16473889

[B44] Pouralibaba H. R. Pérez-De-Luque A. Rubiales D. (2017). Histopathology of the infection on resistant and susceptible lentil accessions by two contrasting pathotypes of *Fusarium oxysporum* f.sp. *lentis*. *Eur. J. Plant Pathol.* 148 53–63. 10.1007/s10658-016-1068-6

[B45] Rispail N. Bani M. Rubiales D. (2015). Resistance reaction of *Medicago truncatula* genotypes to *Fusarium oxysporum*: effect of plant age, substrate and inoculation method. *Crop Pasture Sci.* 66 506–515. 10.1071/cp14216

[B46] Rispail N. Rubiales D. (2014). Identification of sources of quantitative resistance to *Fusarium oxysporum* f. sp. *medicaginis* in *Medicago truncatula*. *Plant Dis.* 98 667–673. 10.1094/pdis-03-13-0217-re 30708554

[B47] Rodríguez-Gálvez E. Mendgen K. (1995). The infection process of *Fusarium oxysporum* in cotton root tips. *Protoplasma* 189 61–72. 10.1007/BF01280291 8580765

[B48] Roncero M. I. G. Hera C. Ruiz-Rubio M. Maceira F. I. G. Madrid M. P. Caracuel Z. (2003). *Fusarium* as a model for studying virulence in soilborne plant pathogens. *Physiol. Mol. Plant Pathol.* 62 87–98. 10.1016/s0885-5765(03)00043-2

[B49] Rubiales D. Fondevilla S. Chen W. Gentzbittel L. Higgins T. J. V. Castillejo M. A. (2015). Achievements and challenges in legume breeding for pest and disease resistance. *Crit. Rev. Plant Sci.* 34 195–236. 10.1080/07352689.2014.898445

[B50] Shi J. Mueller W. C. Beckman C. H. (1992). Vessel occlusion and secretory activities of vessel contact cells in resistant or susceptible cotton plants infected with *Fusarium oxysporum* f. sp. *vasinfectum*. *Physiol. Mol. Plant Pathol.* 40 133–147. 10.1016/0885-5765(92)90040-3

[B51] Tessier B. J. Mueller W. C. Morgham A. T. (1990). Histopathology and ultrastructure of vascular-responses in peas resistant or susceptible to *Fusarium oxysporum* f. sp. *pisi*. *Phytopathology* 80 756–764. 10.1094/Phyto-80-756

[B52] Turrà D. El Ghalid M. Rossi F. Di Pietro A. (2015). Fungal pathogen uses sex pheromone receptor for chemotropic sensing of host plant signals. *Nature* 527 521–524. 10.1038/nature15516 26503056

[B53] Vance C. P. Kirk T. K. Sherwood R. T. (1980). Lignification as a mechanism of disease resistance. *Annu. Rev. Phytopathol.* 18 259–288. 10.1146/annurev.py.18.090180.001355

[B54] Vidhyasekaran P. (2008). *Fungal Pathogenesis in Plants and Crops: Molecular Biology and Host Defense Mechanisms.* Boca Raton, FL: CRC Press.

[B55] Walters D. Newton A. C. Lyon G. (2007). *Induced Resistance for Plant Defence: a Sustainable Approach to Crop Protection.* Oxford: Wiley-Blackwell 10.1002/9780470995983

[B56] Western T. L. Skinner D. J. Haughn G. W. (2000). Differentiation of mucilage secretory cells of the *Arabidopsis* seed coat. *Plant Physiol.* 122 345–355. 10.1104/pp.122.2.345 10677428 PMC58872

[B57] Yadeta K. A. Thomma B. P. H. J. (2013). The xylem as battleground for plant hosts and vascular wilt pathogens. *Front. Plant Sci.* 4:97. 10.3389/fpls.2013.00097 23630534 PMC3632776

[B58] Zvirin T. Herman R. Brotman Y. Denisov Y. Belausov E. Freeman S. (2010). Differential colonization and defence responses of resistant and susceptible melon lines infected by *Fusarium oxysporum* race 1.2. *Plant Pathol.* 59 576–585. 10.1111/j.1365-3059.2009.02225.x

