# Supplementary material for: Physical and Chemical Barriers in Root Tissues Contribute to Quantitative Resistance to Fusarium oxysporum f. sp. pisi in Pea
Source: Front Plant Sci. 2018 Feb 19;9:199. doi: 10.3389/fpls.2018.00199 (PMC5826057; doi:10.3389/fpls.2018.00199)
Supplement: Supplementary file 1 [file Data_Sheet_1.DOCX]

Supplementary Material

Physical and chemical barriers in root tissues contribute to quantitative resistance to Fusarium oxysporum f. sp. pisi in pea

**Bani M.^1,2^, Pérez-De-Luque A.^3^, Rubiales D.^1^, Rispail N.^1,*^**

1 Institute for Sustainable Agriculture–CSIC, Córdoba, Spain

2 Ecole Nationale Supérieure de Biotechnologie, Constantine, Algérie

3 IFAPA, Centro Alameda del Obispo, Área de Mejora y Biotecnología, Córdoba, Spain

***Correspondence:** Rispail N.: [nrispail@ias.csic.es](mailto:nrispail@ias.csic.es)

**Supplementary Figures:**

**Supplementary Figure S1.** Disease severity of *Fop* race 2 on the pea accessions used in this study. The histogram show the final disease severity expressed as percentage of leaves with symptoms estimated after 30 dpi. Vertical bars are standard errors for n= 3. Different letters between each histogram indicates significant difference between values according to Tukey’s range test at α = 0.05. Control: control plants maintained non-inoculated of the P21 susceptible accession.

**Supplementary Figure S2.** Isolation of *Fop* race 2 colonies from inoculated pea plant tissues. Photographs compare the extension of *Fop* race 2 colonies out of plant tissues from resistant (A), partially resistant (B) or susceptible accessions (C) at 7 dpi. R, B, M and A stand for root, basal stem, middle stem and apex sections, respectively.

**Supplementary Figure S3.** Histopathology of *Fop* in resistant accessions. (**A**) TBO- stained cross section of the resistant accession P633 root inoculated with root trimming at 7 dpi showing *Fop* colonization of xylem vessel (Black arrows). (**B**) TBO-stained cross section of the resistant accession P42 inoculated without root trimming at 7 dpi showing the absence of fungal structure in vascular tissue. (**C**) TBO-stained cross section of the hypocotyl of the resistant accessions JI 1760 inoculated with root trimming at 7 dpi showing the absence of *Fop* infection in this tissue. Co, cortex, Fb, fiber, Pc, xylem parenchyma cells, Xy, xylem vessel. Bar = 25 µm.

**
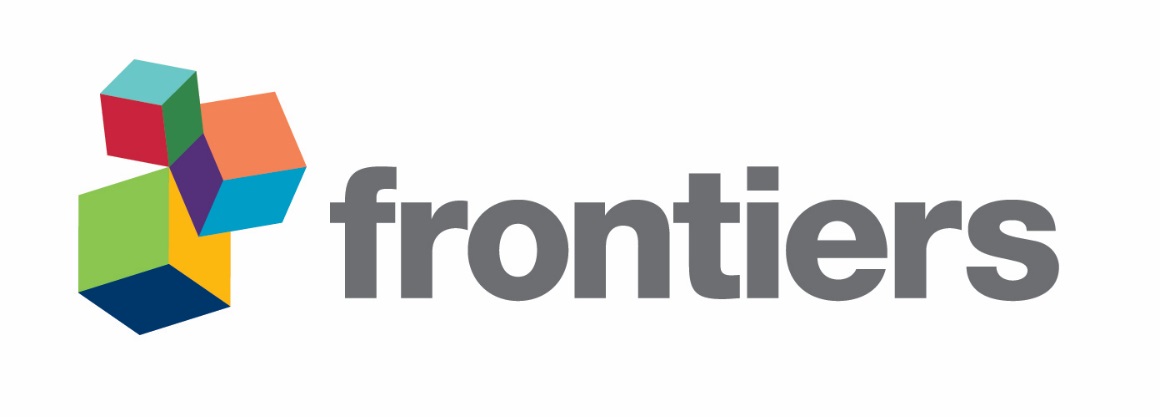
**
